# Association Between Spine Surgery and Availability of Opioid Medication

**DOI:** 10.1001/jamanetworkopen.2020.8974

**Published:** 2020-06-25

**Authors:** Nafisseh S. Warner, Elizabeth B. Habermann, W. Michael Hooten, Andrew C. Hanson, Darrell R. Schroeder, Jennifer L. St. Sauver, Paul M. Huddleston, Mohamad Bydon, Julie L. Cunningham, Halena M. Gazelka, David O. Warner

**Affiliations:** 1 Department of Anesthesiology and Perioperative Medicine, Mayo Clinic, Rochester, Minnesota; 2 Robert D. and Patricia E. Kern Center for the Science of Health Care Delivery, Mayo Clinic, Rochester, Minnesota; 3 Department of Health Sciences Research, Mayo Clinic, Rochester, Minnesota; 4 Department of Orthopedic Surgery, Mayo Clinic, Rochester, Minnesota; 5 Department of Neurological Surgery, Mayo Clinic, Rochester, Minnesota; 6 Department of Pharmacy, Mayo Clinic, Rochester, Minnesota

## Abstract

**Importance:**

Prolonged prescribing of opioids after spine surgery is often perceived as a negative outcome, but successful opioid reduction may occur despite continued prescribing. Improved characterization of opioid availability before and after surgery is necessary to identify these successes.

**Objective:**

To evaluate the association between spine surgery and modification of opioid availability postoperatively by using consistent definitions to classify opioid availability before and after surgery.

**Design, Setting, and Participants:**

This population-based cohort study included 2223 adults (age ≥18 years) who underwent spine surgery in Olmsted County, Minnesota, from January 1, 2005, through December 31, 2016. Data were analyzed from April 1, 2019, to December 1, 2019.

**Exposures:**

Preoperative opioid availability based on prescription data in the 180 days before surgery in accordance with Consortium to Study Opioid Risks and Trends (CONSORT) definitions.

**Main Outcomes and Measures:**

Successful modification of opioid availability, defined as an improvement in CONSORT status postoperatively (assessed from 181 to 365 days after surgery) compared with preoperative status, or continued absence of opioid availability for patients with no preoperative availability. Multivariable logistic regression was used to assess the association between preoperative opioid availability and successful modification by 1 year after undergoing surgery.

**Results:**

Of 2223 patients included in the study, 1214 were male (54.6%), with a median age of 55 years (interquartile range, 43-68) years. Patients were classified as having no (778 [35.0%]), short-term (1118 [50.3%]), episodic (227 [10.2%]), or long-term (100 [4.5%]) preoperative opioid availability. Of the 2148 patients (96.6%) who were alive at 1 year, postoperative opioid availability was classified as no (1583 [73.7%]), short-term (398 [18.5%]), episodic (104 [4.8%]), and long-term (63 [2.9%]). A total of 1672 patients (77.8%) had successful modification of opioid availability, with success of 83.0% for those with no preoperative availability, 74.9% for those with short-term preoperative availability, 79.8% for those with episodic preoperative availability, and 64.4% for those with long-term preoperative opioid availability. In multivariable analysis, success was significantly associated with preoperative opioid availability (odds ratio [OR] for short term, 0.61 [95% CI, 0.48-0.77]; OR for episodic, 0.95 [95% CI, 0.64-1.40]; OR long term, 0.49 [95% CI, 0.30-0.82]; *P* < .001 overall vs no availability).

**Conclusions and Relevance:**

In this study, when following standardized CONSORT definitions, 4 of 5 adults undergoing spine surgery in a population-based cohort met the criteria for a successful pattern of postoperative opioid prescribing. Similar methods to objectively assess changes in opioid prescribing may be clinically useful in other perioperative settings.

## Key Points

**Question:**

Is spine surgery associated with modification of opioid availability postoperatively?

**Findings:**

In this cohort study of 2223 adults undergoing spine surgery, 2148 had complete follow-up through 1 year postoperatively, of whom 77.8% had successful modification of opioid availability. Success was significantly associated with preoperative opioid availability.

**Meaning:**

In this study, when using consistent definitions of long-term opioid availability before and after surgery, most patients experienced successful modification of opioid availability after undergoing spine surgery.

## Teaser

This cohort study evaluates the association of spine surgery with modification of opioid availability postoperatively.

## Introduction

One of the goals of spine surgery for both patients and surgeons is to reduce pain. For patients requiring preoperative prescription opioids for back pain, a related goal is to reduce or eliminate opioid use postoperatively. Many patients do not achieve this goal, with more than 50% continuing to use prescription opioids at 1 year postoperatively (ie, point prevalence opioid use).^1,2,3,4^ Numerous studies^1,5,6,7,8,9,10,11^ have described patient and procedural risk factors for this outcome, including patient age, mental health disorders (ie, depression, anxiety), a history of drug abuse, spinal instrumentation, and preoperative opioid use. Prolonged postoperative opioid use has attracted particular concern in the context of the opioid crisis. If a patient requires prolonged opioid use after surgery, the surgery may be considered to have failed to provide the anticipated improvement in analgesia.

However, a clinical assessment regarding whether surgery was successful in improving pain may benefit from a more nuanced approach to the way in which opioid use is evaluated before and after surgery. For example, a reduction in prescribed opioids postoperatively may be indicative of clinical benefit, even if prescribed opioids are not eliminated completely after surgery. To identify such improvements in opioid prescribing patterns, looking beyond simplified definitions of opioid use (eg, point prevalence at some postoperative time) and using robust yet clinically translatable definitions that can capture the complexity of individual opioid prescribing patterns preoperatively and postoperatively may be necessary. Classification systems such as the Consortium to Study Opioid Risks and Trends (CONSORT) criteria^12,13^ have been widely used to categorize longitudinal patterns of opioid prescribing among those with chronic pain by using standardized definitions at each time point of interest and may also be useful in the perioperative setting. The CONSORT criteria was specifically designed with support from the National Institute on Drug Abuse to improve understanding of longitudinal trends in opioid prescribing among patients with chronic noncancer pain through the assessment of more than 3 million opioid prescribing episodes.

The purpose of this population-based cohort study was to evaluate the association of spine surgery with modifications in postoperative opioid availability, as assessed through the CONSORT classification system, which allows for consistent and comprehensive assessment of longitudinal opioid availability.

## Methods

This population-based cohort study used the resources of the Rochester Epidemiology Project (REP), a unique medical record linkage system that collects and links health care records across health care institutions that serve the population of southeastern Minnesota.^14,15^ Approval was obtained from the institutional review boards of the Mayo Clinic and Olmsted Medical Center, Rochester, Minnesota, which waived requirement for written informed consent because of minimal patient risk. This study followed the Strengthening the Reporting of Observational Studies in Epidemiology (STROBE) reporting guideline.^16^

### Study Patients

All adult patients (age ≥18 years) residing in the 11-county area included in the REP who underwent spine surgery from January 1, 2005, through December 31, 2016, were identified based on *Current Procedural Terminology* codes, as used in previous studies^17^ (eTable 1 in the Supplement). *Current Procedural Terminology* codes were verified through manual review of the medical records for a random sample of 50 study participants, with 1 of us (N.S.W.) verifying procedure codes against procedures documented in the medical record; there were no instances of discordance. Patients who denied research authorization (MN statute 144.295) (n = 143) and patients who did not live to hospital discharge were excluded (n = 0). For patients with multiple procedures during the study period, only the first qualifying procedure was analyzed. To ensure adequate availability of preoperative prescription data, patients who were not residents in the study area for at least 180 days before the surgical procedure were excluded (n = 22).

### Prescription Information and Opioid Use Definitions

All outpatient opioid analgesic prescriptions for the study cohort from 180 days before to 1 year after the index surgical procedure were obtained through REP resources.^14^ Prescriptions were classified according to medication, dosage, and route. Whenever more than 1 prescription was written for a single medication type on a single day, only the last prescription was considered valid because administrative database review revealed that earlier prescriptions given for any medication on the same date represented prescriptions that were later overwritten (ie, invalid). The number of days of available opioids for each prescription was calculated based on the assumption that patients consumed the medication at the maximum prescribed rate. Opioid prescriptions were quantified by calculation of average daily oral morphine milligram equivalents based on the maximum prescribed rate for each unique prescription in accordance with the Centers for Disease Control and Prevention opioid calculation tool.^18^

### Definitions of Prescription Opioid Availability

Preoperative prescription opioid availability was quantified according to CONSORT definitions (eTable 2 in the Supplement).^12^ Patients were categorized as having no opioid availability if they had no opioid prescriptions within 180 days before surgery. For the other categories, the time from the first to last prescription received within the 180 days before surgery was defined as the period of prescribing. If the period of prescribed opioids was fewer than 90 days, the patient was considered to have short-term availability. If the period was 90 days or more, the patients were considered to have long-term availability if they either had more than 10 prescriptions during the prescribing period or if they had opioids available for more than 120 days based on consumption according to the maximum prescribed rate. Patients with a prescribing period of more than 90 days who did not meet the criteria for long-term availability were classified as having episodic availability. Postoperative prescribed opioid availability was also defined in accordance with CONSORT definitions using opioid prescription data available between 181 and 365 days after the date of hospital discharge (ie, no, short-term, episodic, or long-term availability).

### Outcomes

The primary outcome of interest was a pattern of postoperative availability consistent with successful postoperative opioid prescribing, defined as (1) an improvement in CONSORT status 181 to 365 days postoperatively compared with preoperatively (ie, short-term to no availability; episodic to short-term or no availability; or long-term to episodic, short-term, or no availability) or (2) continued absence of opioid use for patients with no preoperative opioid availability. Secondary outcomes included 7-day point prevalence of prescription opioid availability at 90 and 365 days after surgery (ie, any prescription opioid available within 7 days of the time point of interest).

### Statistical Analysis

**Figure 1.**
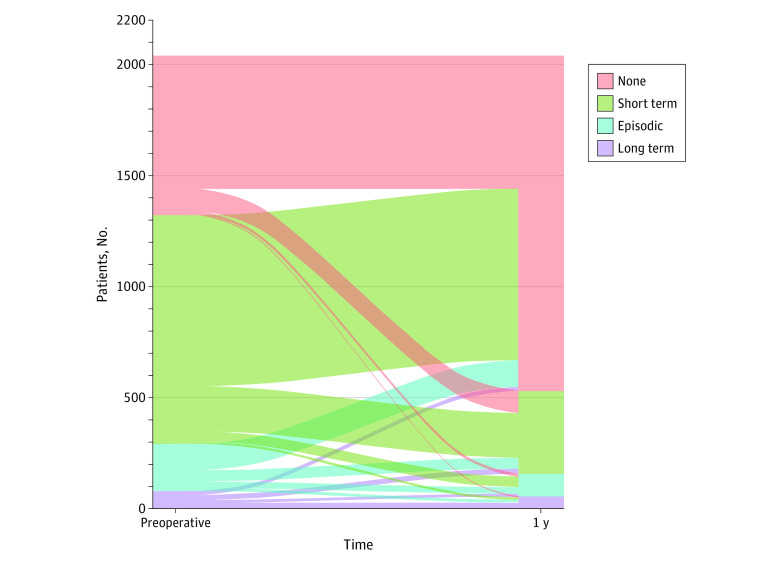
Transition From Preoperative to Postoperative Opioid Availability

**Figure 2.**
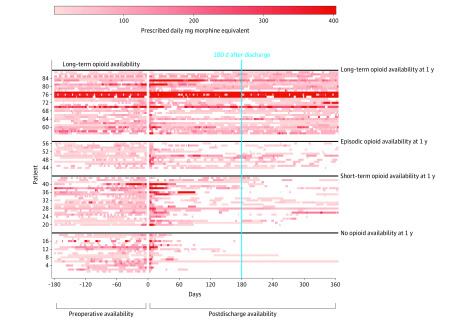
Complete Opioid Prescription Data for Patients With Long-term Preoperative Opioid Availability From 180 Days Through 1 Year Postoperatively Each row represents a unique patient. Each unique colored box represents a new opioid prescription and its daily morphine milligram equivalents, with darker red representing higher morphine milligram equivalents. Patients are clustered according to their Consortium to Study Opioid Risks and Trends (CONSORT) opioid availability classification at 1 year postoperatively. Two patients classified as having no opioid availability at 1 year had a substantial period during the 180 days after discharge in which opioids were available. These patients did not receive any prescriptions for opioids, but each had a previous prescription for tramadol hydrochloride, which provided prolonged opioid availability extending into this period.

Patient, procedural, and hospital characteristics are presented according to preoperative CONSORT definition opioid availability categories as number (percentage) for categorical variables and median (interquartile range [IQR]) for continuous variables. Characteristics were compared across groups using Pearson χ^2^ tests for categorical variables and Kruskal-Wallis rank sum tests for continuous variables. Primary outcomes at 1 year postoperatively are presented as number (percentage) according to preoperative CONSORT definition opioid availability categories. Multivariable logistic regression was used to assess the association between preoperative opioid availability and successful modification in opioid availability at 1 year, with adjustment for potentially confounding variables, including age, sex, spinal instrumentation, Charlson Comorbidity Index, preoperative opioid availability (according to CONSORT definitions), depression, anxiety, tobacco use, and hospital discharge location. Results of the multivariable model are presented as odd ratios (ORs) with 95% CIs. To account for potential variability in prescribing practices over time, the date of the surgical procedure was included in the multivariable model as a restricted cubic spline with 5 knots located at the fifth percentile, lower quartile, median, upper quartile, and 95th percentile of the observed distribution. Patient transition between CONSORT definition categories is displayed visually in an alluvial plot (Figure 1). Opioid availability patterns from 180 days preoperatively to 365 days after hospital discharge are shown for patients with long-term opioid availability preoperatively using patient-specific heat maps (Figure 2). A 2-sided *P* < .05 was considered statistically significant. Data management, summaries, and analyses were done using SAS software, version 9.4 (SAS Institute) and R, version 3.4.1 (R Foundation for Statistical Computing).

## Results

A total of 2223 adult patients (1214 [54.6%] male; median age, 55 years [IQR, 43-68 years]) who resided in an 11-county region of southeastern Minnesota were included (eFigure in the Supplement). Overall, patients were classified according to the CONSORT definitions as having no preoperative opioid availability (778 [35.0%]), short term preoperative opioid availability (1118 [50.3%]), episodic preoperative opioid availability (227 [10.2%]), and long-term (100 [4.5%]) preoperative opioid availability based on the pattern of opioid prescribing in the 180-day period before surgery.

Patient and procedural characteristics are presented in Table 1 according to the CONSORT definitions of preoperative opioid availability. There were differences in sex distributions, with females comprising 304 of 778 patients (39.1%) with no availability, 517 of 1118 (46.2%) with short-term availability, 135 of 227 (59.5%) with episodic availability, and 53 of 100 (53.0%) with long-term availability (*P* < .001). Comorbidity burden also differed by preoperative opioid availability, with high levels of baseline illness (American Society of Anesthesiologists physical status score of 3-4) observed in 228 patients (30.4%) with no availability, 309 (28.5%) with short-term availability, 79 (35.7%) with episodic availability, and 38 (39.2%) with long-term availability (*P* = .04) (Table 1).

**Table 1.** Patient and Procedural Characteristics According to Preoperative CONSORT Opioid Availability^a^

Characteristic	Preoperative opioid availability				*P* value
	None (n = 778)	Short-term (n = 1118)	Episodic (n = 227)	Long-term (n = 100)	
Age, median (IQR), y	56 (43-70)	53 (43-66)	55 (43-68)	55 (41-67)	.10
Sex					
Male	474 (60.9)	601 (53.8)	92 (40.5)	47 (47.0)	<.001
Female	304 (39.1)	517 (46.2)	135 (59.5)	53 (53.0)	
White	710 (91.3)	1047 (93.6)	213 (93.8)	89 (89.0)	.10
ASA physical status score (n = 2150)					.04
1-2	521 (69.6)	774 (71.5)	142 (64.3)	59 (60.8)	
3-4	228 (30.4)	309 (28.5)	79 (35.7)	38 (39.2)	
Charlson Comorbidity Index, median (IQR)	1 (0-3)	1 (0-3)	1 (0-4)	2 (1-5)	<.001
Psychological diagnosis					
Depression	245 (31.5)	426 (38.1)	122 (53.7)	53 (53.0)	<.001
Anxiety	149 (19.2)	232 (20.8)	70 (30.8)	46 (46.0)	<.001
Substance use diagnosis					
Tobacco	199 (25.6)	365 (32.6)	107 (47.1)	58 (58.0)	<.001
Alcoholism	70 (9.0)	110 (9.8)	26 (11.5)	17 (17.0)	.08
Illicit drugs	32 (4.1)	63 (5.6)	20 (8.8)	29 (29.0)	<.001
Procedural characteristics					
Anesthesia duration, median (IQR), min	242 (187-338)	217 (176-280)	225 (186-322)	253 (190-358)	<.001
Surgery duration, median (IQR), min	157 (111-231)	138 (105-190)	148 (111-227)	165 (116-257)	<.001
Instrumentation	274 (35.2)	254 (22.7)	57 (25.1)	34 (34.0)	<.001
Hospital length of stay, median (IQR), d	2 (1-3)	1 (1-3)	2 (1-3)	3 (1-5)	<.001
Discharge location					
Home	678 (87.1)	978 (87.5)	201 (88.5)	77 (77.0)	.003
Not home	91 (11.7)	106 (9.5)	22 (9.7)	20 (20.0)	
Not documented	9 (1.2)	34 (3.0)	4 (1.8)	3 (3.0)	
Total discharge prescription MME, median (IQR) (n = 1934)^b^	525 (375-750)	500 (375-750)	600 (375-900)	750 (525-1250)	<.001
Total supply of discharge prescriptions, median (IQR), d (n = 1934)^b^	7 (5-9)	5 (5- 9)	7 (5-10)	9 (5-13)	<.001

Abbreviations: ASA, American Society of Anesthesiologists; CONSORT, Consortium to Study Opioid Risks and Trends; IQR, interquartile range; MME, oral morphine milligram equivalents.

^a^ Data are presented as number (percentage) of patients unless otherwise indicated. Categorical variables were compared using χ^2^ tests. Continuous variables were compared using Wilcoxon rank sum tests.

^b^ Data were only available for patients discharged home.

The presence of psychiatric and substance abuse diagnoses was also associated with higher preoperative opioid availability. Among patients who had been prescribed opioids in the 180 days before surgery, those with higher opioid availability had longer duration of surgery (medians [IQRs] of 138 [105-190] minutes with short-term availability, 148 [111-227] minutes with episodic availability, and 165 [116-257] minutes with long-term availability) and were more likely to require surgery with instrumentation (254 [22.7%] with short-term availability, 57 [25.1%] with episodic availability, and 34 [34.0%] with long-term availability). Patients with long-term availability preoperatively also had longer lengths of stay (medians [IQRs] of 3 [1-5] days compared with 2 [1-3] days with no availability, 1 [1-3] day with short-term availability, and 2 [1-3] days with episodic availability) and were less likely to be discharged home (ie, more likely to be discharged to a rehabilitation or similar facility) compared with patients in the other categories (77 [77.0%] vs 678 [87.1%] with no availability, 978 [87.5%] with short-term availability, and 201 [88.5%] with episodic availability). Among those discharged home, higher preoperative opioid availability was associated with discharge prescriptions having higher total morphine milligram equivalents (medians [IQRs], 525 [375-750] with no availability, 500 [375-750] with short-term availability, 600 [375-900] with episodic availability, and 750 [525-1250] with long-term availability; *P* < .001). The reoperation rate at 30 days was 3.7% and at 90 days was 6.9%.

Of the 2223 patients, 2148 (96.6%) were alive and still residing in the 11-county catchment area at 1 year after hospital discharge. According to CONSORT definitions applied from 181 to 365 days postoperatively after hospital discharge, of the 2148 patients, 1583 (73.7%) had no postoperative opioid availability, 398 (18.5%) had short-term postoperative opioid availability, 104 (4.8%) had episodic postoperative opioid availability, and 63 (2.9%) had long-term postoperative opioid availability. A total of 1672 patients (77.8%) met the criteria for successful patterns of postoperative opioid prescribing, defined as having improved CONSORT status or no opioid availability 1 year postoperatively for those who had no preoperative availability. Rates of success for preoperative opioid availability were 628 of 757 (83.0%) for no preoperative opioid availability, 810 of 1081 (74.9%) for short-term preoperative opioid availability, 178 of 223 (79.8%) for episodic preoperative opioid availability, and 56 of 87 (64.4%) for long-term preoperative opioid availability (Figure 1 and Table 2). For patients who had long-term opioid availability preoperatively, the daily opioid availability from 180 days before surgery through 365 days following hospital discharge is presented in Figure 2. Within each CONSORT category, there was considerable intraindividual variability.

**Table 2.** Opioid Use Outcomes at 1 Year in Patients With Follow-up Data Available

Outcome	Preoperative CONSORT opioid availability, No. (%)				*P* value
	None (n = 757)	Short-term (n = 1081)	Episodic (n = 223)	Long-term (n = 87)	
7-d Point prevalence availability^a^					
No	726 (95.9)	1007 (93.2)	184 (82.5)	43 (49.4)	<.001
Yes	31 (4.1)	74 (6.8)	39 (17.5)	44 (50.6)	
1-y Postoperative CONSORT opioid availability (n = 2148)					
No (n = 1583)	628 (83.0)	810 (74.9)	126 (56.5)	19 (21.8)	<.001
Short-term (n = 398)	110 (14.5)^b^	212 (19.6)^b^	52 (23.3)	24 (27.6)	
Episodic (n = 104)	13 (1.7)^b^	47 (4.3)^b^	31 (13.9)^b^	13 (14.9)	
Long-term (n = 63)	6 (0.8)^b^	12 (1.1)^b^	14 (6.3)^b^	31 (35.6)^b^	
Success^c^					
No	129 (17.0)	271 (25.1)	45 (20.2)	31 (35.6)	<.001
Yes	628 (83.0)	810 (74.9)	178 (79.8)	56 (64.4)	

Abbreviation: CONSORT, Consortium to Study Opioid Risks and Trends.

^a^ Defined as any prescription opioid availability in the 7 days from 358 to 365 days after hospital discharge.

^b^ Unsuccessful patterns of postoperative opioid availability according to our criteria.

^c^ Success was defined as occurring when a patient was in a lower opioid use classification assessed by the CONSORT definitions from 181 to 365 days postoperatively compared with preoperative status or when a patient met the criteria for no opioid availability.

Of the 2148 patients available for follow-up, 188 (8.8%) had opioids available within the 7 days before 1 year after hospital discharge (ie, point prevalence opioid availability). Point-prevalence opioid availability at 1 year postoperatively was 31 of 757 (4.1%) for those with no preoperative availability, 74 of 1081 (6.8%) for those with short-term preoperative availability, 39 of 223 (17.5%) for those with episodic preoperative availability, and 44 of 87 (50.6%) for those with long-term preoperative availability.

In multivariable analysis, success in postoperative opioid prescribing was significantly associated with preoperative opioid availability (OR, 0.61 [95% CI, 0.48-0.77] for short-term availability; OR, 0.95 [95% CI, 0.64-1.40] for episodic availability; and OR 0.49 [95% CI, 0.30-0.82] for long-term compared with no availability; *P* < .001) (Table 3). Compared with patients with no preoperative opioid availability, those with long-term and short-term preoperative availability but not those with episodic availability were less likely to meet the criteria for success. Other factors independently associated with a lower success rate included a higher burden of comorbidity (OR, 0.45; 95% CI, 0.32-0.63 for Charlson comorbidity index >4 vs reference of 0), the presence of anxiety (OR, 0.76; 95% CI, 0.58-0.99), and not being discharged to home after surgery (OR, 0.66; 95% CI, 0.47-0.94). Success did not depend on age, whether instrumentation was used, tobacco abuse or the presence of depression.

**Table 3.** Multivariable Logistic Model for Successful Opioid Modification at 1 Year^a^

Outcome	Odds ratio (95% CI)	*P* value
Age per 10 y	1.06 (0.98-1.15)	.13
Sex		
Male	1 [Reference]	.10
Female	0.83 (0.67-1.04)	
Instrumentation	0.86 (0.68-1.10)	.22
Charlson Comorbidity Index		
0	1 [Reference]	<.001
1	0.67 (0.50-0.90)	
2-3	0.61 (0.45-0.83)	
≥4	0.45 (0.32-0.63)	
Preoperative opioid availability		
No	1 [Reference]	<.001
Short-term	0.61 (0.48-0.77)	
Episodic	0.95 (0.64-1.40)	
Long-term	0.49 (0.30-0.82)	
Depression	0.89 (0.70-1.13)	.32
Tobacco use	0.84 (0.67-1.05)	.13
Anxiety	0.76 (0.58-0.99)	.04
Discharge location		
Home	1 [Reference]	.06
Not home	0.66 (0.47-0.94)	
Not documented	1.02 (0.50-2.08)	

^a^ Only patients living in the catchment area at 1 year after discharge were included in the analysis (n = 2148). In addition to the covariables presented in the table, to account for variability in prescribing practices across the time frame of the study, the model was adjusted for date of surgery using restricted cubic splines. Model concordance was 0.64, and there was no evidence of significant lack of fit (Hosmer Lemeshow goodness of fit test, *P* = .38).

## Discussion

When patterns of opioid availability were described according to standardized CONSORT definitions during the 180 days before surgery (preoperative) and 181 to 365 days after hospital discharge (postoperative), 77.8% of a population-based cohort of patients undergoing spine surgery met the criteria for a successful pattern of postoperative opioid prescribing. In contrast, if success was defined as no available prescription opioids at 1 year postoperatively (ie, point-prevalence prescription opioid availability), 91.2% of patients met the criteria for success. The latter definition underestimated success in those with long-term preoperative opioid availability and overestimated success in those with short-term or no preoperative availability compared with the former definition.

For most patients with back pain, one of the goals of surgical intervention is to improve pain and reduce or eliminate analgesic use, especially the use of opioids. For patients not using opioids preoperatively, the hope is that opioids will not be required postoperatively for the long term. Determining whether these goals are met requires quantification of preoperative and postoperative opioid prescribing patterns. However, there is little consensus on how to best quantify perioperative opioid prescribing in surgical patients, and several challenges to such quantification. For example, opioid use and prescription opioid availability are distinct entities. The former uses an assessment of consumption, which relies on either patient self-report or direct observation and is difficult to accurately measure in large population-based studies. The latter uses prescription information to estimate the availability of prescribed opioids, which can be reliably ascertained from prescription databases. Several recent publications have highlighted a critical need for standardization of how opioid availability or use is described preoperatively and postoperatively across surgery types.^19,20^ Criteria to define a lack of preoperative opioid availability or use have included no self-reported opioid use or prescription availability at the time of surgery,^1,4,7,21^ no prescription opioid availability between 90 and 7 days before surgery,^22^ no opioid availability in the 180 days before surgery,^5,8,23^ no opioid availability in the 12 months before surgery,^24^ and no opioid availability from 365 to 7 days before surgery.^25^ Some studies report opioid use or availability as a dichotomous variable (yes or no), whereas others analyzed opioid dose typically in oral morphine equivalents.^3,11^ This methodologic heterogeneity makes it difficult to compare studies and to determine whether surgery has been associated with modification of opioid prescribing among those with preoperative opioid availability or with a new pattern of opioid use among those without preoperative availability.

Although there have been several attempts to discriminate patient groups at highest risk for prolonged postoperative opioid use based on the duration and/or dose of preoperative prescribing,^3,6,8,10,11,24^ few studies^5^ have used consistent definitions before and after surgery. This limits the ability to determine associations of preoperative opioid availability with the modification of postoperative availability. The CONSORT framework facilitates robust assessment of longitudinal opioid availability through prescription records, but it remains clinically pragmatic by not necessitating calculations of cumulative opioid doses. In this investigation, we chose to analyze opioid prescription availability patterns in accordance with CONSORT definitions during the 180 days before surgery as a marker of preoperative opioid status. Recognizing that early postoperative opioid availability (ie, within the first months after surgery) may not be reflective of longer-term availability,^7,8,23^ we opted to define postoperative opioid status in accordance with CONSORT definitions assessed between 181 and 365 days postoperatively. Although other intervals could be used, this approach provided a clinically relevant benchmark in the assessment of analgesic outcomes after surgery while also providing an appropriate space for any potential associations of early surgical complications with postoperative opioid use. Through the use of CONSORT or similar classification schemes, a clinician may be able to estimate a patient’s preoperative potential for successful modification of opioid availability after surgery; however, this was not the goal of this investigation and predictive models would need to be developed and validated to provide such patient-level predictions.

Approximately one-third of patients in this cohort had no preoperative opioid availability, consistent with the range of 22% to 53% reported in previous studies (albeit with other definitions of availability).^3,4,5,11,22,24,26^ Also consistent with other studies,^4^ factors associated with greater preoperative opioid availability included female sex, a greater burden of comorbidity, depression, anxiety, and substance dependence. Of interest, there was not a consistent association between preoperative availability and markers of surgical complexity (eg, duration of surgery and the need for instrumentation). Although the varying definitions of preoperative and postoperative opioid availability and heterogeneity in the type of spine procedures analyzed among studies make it difficult to make direct comparisons, the finding that patients who underwent spine surgery with the greatest preoperative opioid availability were at greater risk to have opioids prescribed for an extended period postoperatively is broadly consistent with previous studies.^3,5,8,23^ Our point-prevalence rate of opioid availability at 1 year postoperatively (8.8%) was also consistent with previous work,^23^ as was the high point-prevalence rate specifically in those with the highest opioid availability.^3^ However, our results also demonstrated the limitations of using cross-sectional assessments of postoperative opioid availability to assess the association of surgery with modification of opioid prescribing. For example, although only 4.1% of patients with no preoperative availability had opioids available at 1 year after discharge (point prevalence), 17.0% had at least short-term postoperative availability from 181 to 365 days after discharge, with some experiencing episodic or long-term availability. At the other end of the spectrum, 50.6% of patients with long-term preoperative availability had 1-year point prevalence availability, but 64.4% were successful in reducing opioid availability.

Although the goal of this investigation was not to identify predictors of postoperative opioid use, multivariable analysis revealed that those with more preoperative availability were less likely to experience successful modification of opioid availability, with the exception of those with episodic preoperative availability. The reason for this exception is not clear, although those with episodic availability were less likely to have no opioid availability at year 1 than those with short-term preoperative availability despite approximately one-quarter successfully transitioning from episodic to short-term postoperative availability.

Of interest, some factors that were associated with increased preoperative availability were not independently associated with less postoperative success, including female sex, depression, tobacco use, and the need for instrumentation. If the latter is considered as an indicator of surgical complexity, this finding suggests that patients undergoing more complex spine surgical procedures are not at increased risk for an unsuccessful pattern of postoperative opioid use. Again, direct comparisons with other studies are problematic, but because many of these factors have been found to be risk factors for prolonged opioid use after spine surgery,^1,21,23^ this finding provides another example of how point-prevalence approaches to analyzing postoperative opioid availability may be misleading in identifying patients at risk for not having success in modifying preoperative opioid availability.^27^

### Strengths and Limitations

The strengths of this investigation include using a population-based cohort (thus minimizing referral bias) with complete ascertainment of opioid prescription data and full access to medical records of multiple institutions through an established medical records linkage system.

This study has limitations. Interpretation of prescription data in terms of availability on a given date assumes that all prescriptions were filled and that opioids were used at the prescribed rate; actual availability on a given day may be less (eg, if the prescription was not filled) or more (if opioids were used more often than the prescribed rate). It is also possible that patients could have obtained opioid prescriptions not captured by our data set, although previous validation work with these data suggests that this is minimal. Although we adjusted our statistical models for many of the factors that have previously been identified as being associated with postoperative opioid use, associations may be confounded by other relevant unmeasured variables. Indications for opioid prescribing were not available, and it is possible that some prescriptions were not related to the index surgical procedure. In addition, these data were derived from a relatively demographically and socioeconomically homogeneous population in southeastern Minnesota, and the generalizability of study findings to other populations is unclear.

## Conclusions

We presented a method to define successful modification of opioid availability after spine surgery as quantified by the CONSORT classification system. Approximately 4 of 5 patients in a geographically defined population demonstrated successful modification of prescribing patterns, with patients who received long-term prescribed opioids preoperatively being least likely to achieve success. Success rates were not congruent with simplified 1-year point prevalence evaluations of opioid availability. Use of CONSORT or similar methods to objectively assess changes in opioid status may be clinically useful in other perioperative settings.

## Notes

Supplement. **eTable 1.** Included CPT Codes and Their Description **eTable 2.** Consortium to Study Opioid Risks and Trends (CONSORT) Definitions for Opioid Availability **eFigure.** Study Population Flow Diagram

## References

[1] Armaghani SJ, Lee DS, Bible JE, Preoperative opioid use and its association with perioperative opioid demand and postoperative opioid independence in patients undergoing spine surgery. Spine (Phila Pa 1976). 2014;39(25):E1524-E1530. doi:10.1097/BRS.0000000000000622 25417827

[2] Jain N, Phillips FM, Weaver T, Khan SN Preoperative chronic opioid therapy: a risk factor for complications, readmission, continued opioid use and increased costs after one- and two-level posterior lumbar fusion. Spine (Phila Pa 1976). 2018;43(19):1331-1338. doi:10.1097/BRS.0000000000002609 29561298

[3] Oleisky ER, Pennings JS, Hills J, Comparing different chronic preoperative opioid use definitions on outcomes after spine surgery. Spine J. 2019;19(6):984-994. doi:10.1016/j.spinee.2018.12.014 30611889

[4] Dunn LK, Yerra S, Fang S, Incidence and risk factors for chronic postoperative opioid use after major spine surgery: a cross-sectional study with longitudinal outcome. Anesth Analg. 2018;127(1):247-254. doi:10.1213/ANE.0000000000003338 29570151 PMC6487073

[5] Schoenfeld AJ, Nwosu K, Jiang W, Risk factors for prolonged opioid use following spine surgery, and the association with surgical intensity, among opioid-naive patients. J Bone Joint Surg Am. 2017;99(15):1247-1252. doi:10.2106/JBJS.16.01075 28763410

[6] Connolly J III, Javed Z, Raji MA, Chan W, Kuo Y-F, Baillargeon J Predictors of long-term opioid use following lumbar fusion surgery. Spine (Phila Pa 1976). 2017;42(18):1405-1411. doi:10.1097/BRS.0000000000002133 28263225 PMC5582019

[7] Lee D, Armaghani S, Archer KR, Preoperative opioid use as a predictor of adverse postoperative self-reported outcomes in patients undergoing spine surgery. J Bone Joint Surg Am. 2014;96(11):e89. doi:10.2106/JBJS.M.00865 24897746

[8] Deyo RA, Hallvik SE, Hildebran C, Use of prescription opioids before and after an operation for chronic pain (lumbar fusion surgery). Pain. 2018;159(6):1147-1154. doi:10.1097/j.pain.0000000000001202 29521813 PMC5955818

[9] Kalakoti P, Hendrickson NR, Bedard NA, Pugely AJ Opioid utilization following lumbar arthrodesis: trends and factors associated with long-term use. Spine (Phila Pa 1976). 2018;43(17):1208-1216. doi:10.1097/BRS.0000000000002734 30045343

[10] Hills JM, Pennings JS, Archer KR, Preoperative opioids and 1-year patient-reported outcomes after spine surgery. Spine (Phila Pa 1976). 2019;44(12):887-895. doi:10.1097/BRS.0000000000002964 30601356

[11] Anderson JT, Haas AR, Percy R, Woods ST, Ahn UM, Ahn NU Chronic opioid therapy after lumbar fusion surgery for degenerative disc disease in a workers’ compensation setting. Spine (Phila Pa 1976). 2015;40(22):1775-1784. doi:10.1097/BRS.0000000000001054 26192725

[12] Von Korff M, Saunders K, Thomas Ray G, De facto long-term opioid therapy for noncancer pain [published correction appears in *Clin J Pain*. 2014;30(9):830]. Clin J Pain. 2008;24(6):521-527. doi:10.1097/AJP.0b013e318169d03b 18574361 PMC3286630

[13] Thiels CA, Habermann EB, Hooten WM, Jeffery MM Chronic use of tramadol after acute pain episode: cohort study. BMJ. 2019;365:l1849. doi:10.1136/bmj.l1849 31088782 PMC6514531

[14] Rocca WA, Yawn BP, St Sauver JL, Grossardt BR, Melton LJ III History of the Rochester Epidemiology Project: half a century of medical records linkage in a US population. Mayo Clin Proc. 2012;87(12):1202-1213. doi:10.1016/j.mayocp.2012.08.012 23199802 PMC3541925

[15] Melton LJ III History of the Rochester Epidemiology Project. Mayo Clin Proc. 1996;71(3):266-274. doi:10.4065/71.3.266 8594285

[16] von Elm E, Altman DG, Egger M, Pocock SJ, Gøtzsche PC, Vandenbroucke JP; STROBE Initiative The Strengthening the Reporting of Observational Studies in Epidemiology (STROBE) statement: guidelines for reporting observational studies. J Clin Epidemiol. 2008;61(4):344-349. doi:10.1016/j.jclinepi.2007.11.008 18313558

[17] Kerezoudis P, Alvi MA, Spinner RJ, Meyer FB, Habermann EB, Bydon M Predictors of unplanned returns to the operating room within 30 days in neurosurgery: insights from a national surgical registry. World Neurosurg. 2019;123:e348-e370. doi:10.1016/j.wneu.2018.11.171 30500576

[18] Centers for Disease Control and Prevention Opioid overdose. Guideline overview. Published 2019 Accessed January 7, 2019. https://www.cdc.gov/drugoverdose/prescribing/guideline.html#anchor_1561563251

[19] Wu CL, King AB, Geiger TM, ; Fourth Perioperative Quality Initiative Workgroup American Society for Enhanced Recovery and Perioperative Quality Initiative joint consensus statement on perioperative opioid minimization in opioid-naive patients. Anesth Analg. 2019;129(2):567-577. doi:10.1213/ANE.0000000000004194 31082966 PMC7261519

[20] Edwards DA, Hedrick TL, Jayaram J, ; POQI-4 Working Group American Society for Enhanced Recovery and Perioperative Quality Initiative joint consensus statement on perioperative management of patients on preoperative opioid therapy. Anesth Analg. 2019;129(2):553-566. doi:10.1213/ANE.0000000000004018 30768461

[21] Wright AK, Sikora M, Leveque J-C Characterizing the risk of long-term opioid utilization in patients undergoing lumbar spine surgery. Spine (Phila Pa 1976). 2020;45(1):E54-E60. doi:10.1097/BRS.0000000000003199 31415465

[22] Harris AB, Marrache M, Jami M, Chronic opioid use following anterior cervical discectomy and fusion surgery for degenerative cervical pathology. Spine J. Published online September 16, 2019. 2020;20(1):78-86. doi:10.1016/j.spinee.2019.09.011 31536805

[23] Adogwa O, Davison MA, Vuong VD, Reduction in narcotic use after lumbar decompression and fusion in patients with symptomatic lumbar stenosis or spondylolisthesis. Global Spine J. 2019;9(6):598-606. doi:10.1177/2192568218814235 31448192 PMC6693064

[24] Schoenfeld AJ, Belmont PJ Jr, Blucher JA, Sustained preoperative opioid use is a predictor of continued use following spine surgery. J Bone Joint Surg Am. 2018;100(11):914-921. doi:10.2106/JBJS.17.00862 29870441

[25] Harbaugh CM, Lee JS, Chua KP, Association between long-term opioid use in family members and persistent opioid use after surgery among adolescents and young adults. JAMA Surg. 2019;154(4):e185838. doi:10.1001/jamasurg.2018.5838 30810738 PMC6484797

[26] Armaghani SJ, Even JL, Zern EK, Braly BA, Kang JD, Devin CJ The evaluation of donor site pain after harvest of tricortical anterior iliac crest bone graft for spinal surgery: a prospective study. Spine (Phila Pa 1976). 2016;41(4):E191-E196. doi:10.1097/BRS.0000000000001201 26571154

[27] Legislature M Office of the Revisor of Statutes. 2019 Minnesota Statutes. Published 2019. Accessed April 1, 2019. https://www.revisor.mn.gov/statutes/cite/144.295

