# Supplementary material for: Association Between Spine Surgery and Availability of Opioid Medication
Source: JAMA Netw Open. 2020 Jun 25;3(6):e208974. doi: 10.1001/jamanetworkopen.2020.8974 (PMC7317600; doi:10.1001/jamanetworkopen.2020.8974)
Supplement: Supplement. — eTable 1. Included CPT Codes and Their Description eTable 2. Consortium to Study Opioid Risks and Trends (CONSORT) Definitions for Opioid Availability eFigure. Study Population Flow Diagram [file jamanetwopen-3-e208974-s001.pdf]

Supplementary Online Content

Warner NS, Habermann EB, Hooten WM et al. Association between spine surgery and availability of opioid medication. *JAMA Netw Open*. 2020;3(6):e208974. doi:10.1001/jamanetworkopen.2020.8974

eTable 1. Included CPT Codes and Their Description

eTable 2. Consortium to Study Opioid Risks and Trends (CONSORT) Definitions for Opioid Availability

eFigure. Study Population Flow Diagram

This supplementary material has been provided by the authors to give readers additional information about their work.

© 2020 Warner et al. *JAMA Network Open.*

eTable 1. Included CPT Codes and Their Description

Code	Description
22532	Arthrodesis, lateral extracavitary technique, including minimal discectomy to prepare interspace (other than for decompression); thoracic
22533	Arthrodesis, lateral extracavitary technique, including minimal discectomy to prepare interspace (other than for decompression); lumbar
22534	Arthrodesis, lateral extracavitary technique, including minimal discectomy to prepare interspace (other than for decompression); thoracic or lumbar, each additional vertebral segment (List separately in addition to code for primary procedure)
22551	Arthrodesis, anterior interbody, including disc space preparation, discectomy, osteophytectomy and decompression of spinal cord and/or nerve roots; cervical below C2
22552	Arthrodesis, anterior interbody, including disc space preparation, discectomy, osteophytectomy and decompression of spinal cord and/or nerve roots; cervical below C2, each additional interspace (List separately in addition to code for separate procedure)
22554	Arthrodesis, anterior interbody technique, including minimal discectomy to prepare interspace (other than for decompression); cervical below C2
22556	Arthrodesis, anterior interbody technique, including minimal discectomy to prepare interspace (other than for decompression); thoracic
22558	Arthrodesis, anterior interbody technique, including minimal discectomy to prepare interspace (other than for decompression); lumbar
22585	Arthrodesis, anterior interbody technique, including minimal discectomy to prepare interspace (other than for decompression); each additional interspace (List separately in addition to code for primary procedure)
22600	Arthrodesis, posterior or posterolateral technique, single level; cervical below C2 segment
22610	Arthrodesis, posterior or posterolateral technique, single level; thoracic (with lateral transverse technique, when performed)
22612	Arthrodesis, posterior or posterolateral technique, single level; lumbar (with lateral transverse technique, when performed)
22614	Arthrodesis, posterior or posterolateral technique, single level; each additional vertebral segment (List separately in addition to code for primary procedure)
22630	Arthrodesis, posterior interbody technique, including laminectomy and/or discectomy to prepare interspace (other than for decompression), single interspace; lumbar
22632	Arthrodesis, posterior interbody technique, including laminectomy and/or discectomy to prepare interspace (other than for decompression), single interspace; each additional interspace (List separately in addition to code for primary procedure)

© 2020 Warner et al. *JAMA Network Open.*

22633	Arthrodesis, combined posterior or posterolateral technique with posterior interbody technique including laminectomy and/or discectomy sufficient to prepare interspace (other than for decompression), single interspace and segment; lumbar
22634	Arthrodesis, combined posterior or posterolateral technique with posterior interbody technique including laminectomy and/or discectomy sufficient to prepare interspace (other than for decompression), single interspace and segment; each additional interspace and segment (List separately in addition to code for primary procedure)
22840	Posterior non-segmental instrumentation (e.g., Harrington rod technique, pedicle fixation across 1 interspace, atlantoaxial transarticular screw fixation, sublaminar wiring at C1, facet screw fixation) (List separately in addition to code for primary procedure)
22841	Internal spinal fixation by wiring of spinous processes (List separately in addition to code for primary procedure)
22842	Posterior segmental instrumentation (e.g., pedicle fixation, dual rods with multiple hooks and sublaminar wires); 3 to 6 vertebral segments (List separately in addition to code for primary procedure)
22843	Posterior segmental instrumentation (e.g., pedicle fixation, dual rods with multiple hooks and sublaminar wires); 7 to 12 vertebral segments (List separately in addition to code for primary procedure)
22844	Posterior segmental instrumentation (e.g., pedicle fixation, dual rods with multiple hooks and sublaminar wires); 13 or more vertebral segments (List separately in addition to code for primary procedure)
22845	Anterior instrumentation; 2 to 3 vertebral segments (List separately in addition to code for primary procedure)
22846	Anterior instrumentation; 4 to 7 vertebral segments (List separately in addition to code for primary procedure)
22847	Anterior instrumentation; 8 or more vertebral segments (List separately in addition to code for primary procedure)
22848	Pelvic fixation (attachment of caudal end of instrumentation to pelvic bony structures) other than sacrum (List separately in addition to code for primary procedure)
22856	Total disc arthroplasty (artificial disc), anterior approach, including discectomy with end plate preparation (includes osteophytectomy for nerve root or spinal cord decompression and microdissection); single interspace, cervical
22861	Revision including replacement of total disc arthroplasty (artificial disc), anterior approach, single interspace; cervical
63001	Laminectomy with exploration and/or decompression of spinal cord and/or cauda equina, without facetectomy, foraminotomy or discectomy (e.g., spinal stenosis), 1 or 2 vertebral segments; cervical
63003	Laminectomy with exploration and/or decompression of spinal cord and/or cauda equina, without facetectomy, foraminotomy or discectomy (e.g., spinal stenosis), 1 or 2 vertebral segments; thoracic
63005	Laminectomy with exploration and/or decompression of spinal cord and/or cauda equina, without facetectomy, foraminotomy or discectomy (e.g., spinal stenosis), 1 or 2 vertebral segments; lumbar, except for spondylolisthesis
63011	Laminectomy with exploration and/or decompression of spinal cord and/or cauda equina, without facetectomy, foraminotomy or discectomy (e.g., spinal stenosis), 1 or 2 vertebral segments; sacral
63012	Laminectomy with removal of abnormal facets and/or pars inter-articularis with decompression of cauda equina and nerve roots for spondylolisthesis, lumbar (Gill type procedure)

© 2020 Warner et al. *JAMA Network Open.*

63015	Laminectomy with exploration and/or decompression of spinal cord and/or cauda equina, without facetectomy, foraminotomy or discectomy (e.g., spinal stenosis), more than 2 vertebral segments; cervical
63016	Laminectomy with exploration and/or decompression of spinal cord and/or cauda equina, without facetectomy, foraminotomy or discectomy (e.g., spinal stenosis), more than 2 vertebral segments; thoracic
63017	Laminectomy with exploration and/or decompression of spinal cord and/or cauda equina, without facetectomy, foraminotomy or discectomy (e.g., spinal stenosis), more than 2 vertebral segments; lumbar
63020	Laminotomy (hemilaminectomy), with decompression of nerve root(s), including partial facetectomy, foraminotomy and/or excision of herniated intervertebral disc; 1 interspace, cervical
63030	Laminotomy (hemilaminectomy), with decompression of nerve root(s), including partial facetectomy, foraminotomy and/or excision of herniated intervertebral disc; 1 interspace, lumbar
63035	Laminotomy (hemilaminectomy), with decompression of nerve root(s), including partial facetectomy, foraminotomy and/or excision of herniated intervertebral disc; each additional interspace, cervical or lumbar (List separately in addition to code for primary procedure)
63040	Laminotomy (hemilaminectomy), with decompression of nerve root(s), including partial facetectomy, foraminotomy and/or excision of herniated intervertebral disc, reexploration, single interspace; cervical
63042	Laminotomy (hemilaminectomy), with decompression of nerve root(s), including partial facetectomy, foraminotomy and/or excision of herniated intervertebral disc, reexploration, single interspace; lumbar
63043	Laminotomy (hemilaminectomy), with decompression of nerve root(s), including partial facetectomy, foraminotomy and/or excision of herniated intervertebral disc, reexploration, single interspace; each additional cervical interspace (List separately in addition to code for primary procedure)
63044	Laminotomy (hemilaminectomy), with decompression of nerve root(s), including partial facetectomy, foraminotomy and/or excision of herniated intervertebral disc, reexploration, single interspace; each additional lumbar interspace (List separately in addition to code for primary procedure)
63045	Laminectomy, facetectomy and foraminotomy (unilateral or bilateral with decompression of spinal cord, cauda equina and/or nerve root[s], [e.g., spinal or lateral recess stenosis]), single vertebral segment; cervical
63046	Laminectomy, facetectomy and foraminotomy (unilateral or bilateral with decompression of spinal cord, cauda equina and/or nerve root[s], [e.g., spinal or lateral recess stenosis]), single vertebral segment; thoracic
63047	Laminectomy, facetectomy and foraminotomy (unilateral or bilateral with decompression of spinal cord, cauda equina and/or nerve root[s], [e.g., spinal or lateral recess stenosis]), single vertebral segment; lumbar
63048	Laminectomy, facetectomy and foraminotomy (unilateral or bilateral with decompression of spinal cord, cauda equina and/or nerve root[s], [e.g., spinal or lateral recess stenosis]), single vertebral segment; each additional segment, cervical, thoracic, or lumbar (List separately in addition to code for primary procedure)
63050	Laminoplasty, cervical, with decompression of the spinal cord, 2 or more vertebral segments;

© 2020 Warner et al. *JAMA Network Open.*

63051	Laminoplasty, cervical, with decompression of the spinal cord, 2 or more vertebral segments; with reconstruction of the posterior bony elements (including the application of bridging bone graft and non-segmental fixation devices [e.g., wire, suture, mini-plates], when performed)
63055	Transpedicular approach with decompression of spinal cord, equina and/or nerve root(s) (e.g., herniated intervertebral disc), single segment; thoracic
63056	Transpedicular approach with decompression of spinal cord, equina and/or nerve root(s) (e.g., herniated intervertebral disc), single segment; lumbar (including transfacet, or lateral extraforaminal approach) (e.g., far lateral herniated intervertebral disc)
63057	Transpedicular approach with decompression of spinal cord, equina and/or nerve root(s) (e.g., herniated intervertebral disc), single segment; each additional segment, thoracic or lumbar (List separately in addition to code for primary procedure)
63064	Costovertebral approach with decompression of spinal cord or nerve root(s) (e.g., herniated intervertebral disc), thoracic; single segment
63066	Costovertebral approach with decompression of spinal cord or nerve root(s) (e.g., herniated intervertebral disc), thoracic; each additional segment (List separately in addition to code for primary procedure)
63075	Discectomy, anterior, with decompression of spinal cord and/or nerve root(s), including osteophytectomy; cervical, single interspace
63076	Discectomy, anterior, with decompression of spinal cord and/or nerve root(s), including osteophytectomy; cervical, each additional interspace (List separately in addition to code for primary procedure)
63077	Discectomy, anterior, with decompression of spinal cord and/or nerve root(s), including osteophytectomy; thoracic, single interspace
63078	Discectomy, anterior, with decompression of spinal cord and/or nerve root(s), including osteophytectomy; thoracic, each additional interspace (List separately in addition to code for primary procedure)
63081	Vertebral corpectomy (vertebral body resection), partial or complete, anterior approach with decompression of spinal cord and/or nerve root(s); cervical, single segment
63082	Vertebral corpectomy (vertebral body resection), partial or complete, anterior approach with decompression of spinal cord and/or nerve root(s); cervical, each additional segment (List separately in addition to code for primary procedure)
63085	Vertebral corpectomy (vertebral body resection), partial or complete, transthoracic approach with decompression of spinal cord and/or nerve root(s); thoracic, single segment
63086	Vertebral corpectomy (vertebral body resection), partial or complete, transthoracic approach with decompression of spinal cord and/or nerve root(s); thoracic, each additional segment (List separately in addition to code for primary procedure)
63087	Vertebral corpectomy (vertebral body resection), partial or complete, combined thoracolumbar approach with decompression of spinal cord, cauda equina or nerve root(s), lower thoracic or lumbar; single segment

© 2020 Warner et al. *JAMA Network Open.*

63088	Vertebral corpectomy (vertebral body resection), partial or complete, combined thoracolumbar approach with decompression of spinal cord, cauda equina or nerve root(s), lower thoracic or lumbar; each additional segment (List separately in addition to code for primary procedure)
63090	Vertebral corpectomy (vertebral body resection), partial or complete, transperitoneal or retroperitoneal approach with decompression of spinal cord, cauda equina or nerve root(s), lower thoracic, lumbar, or sacral; single segment
63091	Vertebral corpectomy (vertebral body resection), partial or complete, transperitoneal or retroperitoneal approach with decompression of spinal cord, cauda equina or nerve root(s), lower thoracic, lumbar, or sacral; each additional segment (List separately in addition to code for primary procedure)
63101	Vertebral corpectomy (vertebral body resection), partial or complete, lateral extracavitary approach with decompression of spinal cord and/or nerve root(s) (e.g., for tumor or retropulsed bone fragments); thoracic, single segment
63102	Vertebral corpectomy (vertebral body resection), partial or complete, lateral extracavitary approach with decompression of spinal cord and/or nerve root(s) (e.g., for tumor or retropulsed bone fragments); lumbar, single segment
63103	Vertebral corpectomy (vertebral body resection), partial or complete, lateral extracavitary approach with decompression of spinal cord and/or nerve root(s) (e.g., for tumor or retropulsed bone fragments); thoracic or lumbar, each additional segment (List separately in addition to code for primary procedure)
63265	Laminectomy for excision or evacuation of intraspinal lesion other than neoplasm, extradural; cervical
63266	Laminectomy for excision or evacuation of intraspinal lesion other than neoplasm, extradural; thoracic
63267	Laminectomy for excision or evacuation of intraspinal lesion other than neoplasm, extradural; lumbar
63268	Laminectomy for excision or evacuation of intraspinal lesion other than neoplasm, extradural; sacral
63270	Laminectomy for excision of intraspinal lesion other than neoplasm, intradural; cervical
63271	Laminectomy for excision of intraspinal lesion other than neoplasm, intradural; thoracic
63272	Laminectomy for excision of intraspinal lesion other than neoplasm, intradural; lumbar
63273	Laminectomy for excision of intraspinal lesion other than neoplasm, intradural; sacral
63275	Laminectomy for biopsy/excision of intraspinal neoplasm; extradural, cervical
63276	Laminectomy for biopsy/excision of intraspinal neoplasm; extradural, thoracic
63277	Laminectomy for biopsy/excision of intraspinal neoplasm; extradural, lumbar
63278	Laminectomy for biopsy/excision of intraspinal neoplasm; extradural, sacral
63280	Laminectomy for biopsy/excision of intraspinal neoplasm; intradural, extramedullary, cervical
63281	Laminectomy for biopsy/excision of intraspinal neoplasm; intradural, extramedullary, thoracic
63282	Laminectomy for biopsy/excision of intraspinal neoplasm; intradural, extramedullary, lumbar
63283	Laminectomy for biopsy/excision of intraspinal neoplasm; intradural, sacral
63285	Laminectomy for biopsy/excision of intraspinal neoplasm; intradural, intramedullary, cervical
63286	Laminectomy for biopsy/excision of intraspinal neoplasm; intradural, intramedullary, thoracic

© 2020 Warner et al. *JAMA Network Open.*

63287	Laminectomy for biopsy/excision of intraspinal neoplasm; intradural, intramedullary, thoracolumbar
63290	Laminectomy for biopsy/excision of intraspinal neoplasm; combined extradural-intradural lesion, any level
63295	Osteoplastic reconstruction of dorsal spinal elements, following primary intraspinal procedure (List separately in addition to code for primary procedure)
63300	Vertebral corpectomy (vertebral body resection), partial or complete, for excision of intraspinal lesion, single segment; extradural, cervical
63301	Vertebral corpectomy (vertebral body resection), partial or complete, for excision of intraspinal lesion, single segment; extradural, thoracic by transthoracic approach
63302	Vertebral corpectomy (vertebral body resection), partial or complete, for excision of intraspinal lesion, single segment; extradural, thoracic by thoracolumbar approach
63303	Vertebral corpectomy (vertebral body resection), partial or complete, for excision of intraspinal lesion, single segment; extradural, lumbar or sacral by transperitoneal or retroperitoneal approach
63304	Vertebral corpectomy (vertebral body resection), partial or complete, for excision of intraspinal lesion, single segment; intradural, cervical
63305	Vertebral corpectomy (vertebral body resection), partial or complete, for excision of intraspinal lesion, single segment; intradural, thoracic by transthoracic approach
63306	Vertebral corpectomy (vertebral body resection), partial or complete, for excision of intraspinal lesion, single segment; intradural, thoracic by thoracolumbar approach
63307	Vertebral corpectomy (vertebral body resection), partial or complete, for excision of intraspinal lesion, single segment; intradural, lumbar or sacral by transperitoneal or retroperitoneal approach
63308	Vertebral corpectomy (vertebral body resection), partial or complete, for excision of intraspinal lesion, single segment; each additional segment (List separately in addition to codes for single segment)
22100	Partial excision of posterior vertebral component (e.g., spinous process, lamina or facet) for intrinsic bony lesion, single vertebral segment; cervical
22101	Partial excision of posterior vertebral component (e.g., spinous process, lamina or facet) for intrinsic bony lesion, single vertebral segment; thoracic
22102	Partial excision of posterior vertebral component (e.g., spinous process, lamina or facet) for intrinsic bony lesion, single vertebral segment; lumbar
22103	Partial excision of posterior vertebral component (e.g., spinous process, lamina or facet) for intrinsic bony lesion, single vertebral segment; each additional segment (List separately in addition to code for primary procedure)
22105	Partial resection of vertebral component for tumor (e.g., partial facetectomy, without primary grafting); cervical
22106	Partial resection of vertebral component for tumor (e.g., partial facetectomy, without primary grafting); thoracic
22107	Partial resection of vertebral component for tumor (e.g., partial facetectomy, without primary grafting); lumbar

© 2020 Warner et al. *JAMA Network Open.*

22110	Partial excision of vertebral body, for intrinsic bony lesion, without decompression of spinal cord or nerve root(s), single vertebral segment; cervical
22112	Partial excision of vertebral body, for intrinsic bony lesion, without decompression of spinal cord or nerve root(s), single vertebral segment; thoracic
22114	Partial excision of vertebral body, for intrinsic bony lesion, without decompression of spinal cord or nerve root(s), single vertebral segment; lumbar
22116	Partial excision of vertebral body, for intrinsic bony lesion, without decompression of spinal cord or nerve root(s), single vertebral segment; each additional vertebral segment (List separately in addition to code for primary procedure)
22140	Reconstruction of spine with bone graft (autograft, allograft) and/or methylmethacrylate following resection of single vertebral body; cervical
22141	Reconstruction of spine with bone graft (autograft, allograft) and/or methylmethacrylate following resection of single vertebral body; thoracic
22142	Reconstruction of spine with bone graft (autograft, allograft) and/or methylmethacrylate following resection of single vertebral body; lumbar
22145	Reconstruction of spine following vertebral body resection, each additional vertebral body
22206	Osteotomy of spine, posterior or posterolateral approach, 3 columns, 1 vertebral segment (e.g., pedicle/vertebral body subtraction); thoracic
22207	Osteotomy of spine, posterior or posterolateral approach, 3 columns, 1 vertebral segment (e.g., pedicle/vertebral body subtraction); lumbar
22208	Osteotomy of spine, posterior or posterolateral approach, 3 columns, 1 vertebral segment (e.g., pedicle/vertebral body subtraction); each additional vertebral segment (List separately in addition to code for primary procedure)
22210	Osteotomy of spine, posterior or posterolateral approach, 1 vertebral segment; cervical
22212	Osteotomy of spine, posterior or posterolateral approach, 1 vertebral segment; thoracic
22214	Osteotomy of spine, posterior or posterolateral approach, 1 vertebral segment; lumbar
22216	Osteotomy of spine, posterior or posterolateral approach, 1 vertebral segment; each additional vertebral segment (List separately in addition to primary procedure)
22220	Osteotomy of spine, including discectomy, anterior approach, single vertebral segment; cervical
22222	Osteotomy of spine, including discectomy, anterior approach, single vertebral segment; thoracic
22224	Osteotomy of spine, including discectomy, anterior approach, single vertebral segment; lumbar
22226	Osteotomy of spine, including discectomy, anterior approach, single vertebral segment; each additional vertebral segment (List separately in addition to code for primary procedure)
22318	Open treatment and/or reduction of odontoid fracture(s) and or dislocation(s) (including os odontoideum), anterior approach, including placement of internal fixation; without grafting

© 2020 Warner et al. *JAMA Network Open.*

22319	Open treatment and/or reduction of odontoid fracture(s) and or dislocation(s) (including os odontoideum), anterior approach, including placement of internal fixation; with grafting
22325	Open treatment and/or reduction of vertebral fracture(s) and/or dislocation(s), posterior approach, 1 fractured vertebra or dislocated segment; lumbar
22326	Open treatment and/or reduction of vertebral fracture(s) and/or dislocation(s), posterior approach, 1 fractured vertebra or dislocated segment; cervical
22327	Open treatment and/or reduction of vertebral fracture(s) and/or dislocation(s), posterior approach, 1 fractured vertebra or dislocated segment; thoracic
22328	Open treatment and/or reduction of vertebral fracture(s) and/or dislocation(s), posterior approach, 1 fractured vertebra or dislocated segment; each additional fractured vertebra or dislocated segment (List separately in addition to code for primary procedure)
22548	Arthrodesis, anterior transoral or extraoral technique, clivus-C1-C2 (atlas-axis), with or without excision of odontoid process
22586	Arthrodesis, pre-sacral interbody technique, including disc space preparation, discectomy, with posterior instrumentation, with image guidance, includes bone graft when performed, L5-S1 interspace
22590	Arthrodesis, posterior technique, craniocervical (occiput-C2)
22595	Arthrodesis, posterior technique, atlas-axis (C1-C2)
22625	Arthrodesis, lateral transverse process technique, with local bone or bone allograft and/or internal wire fixation, lumbar
22800	Arthrodesis, posterior, for spinal deformity, with or without cast; up to 6 vertebral segments
22802	Arthrodesis, posterior, for spinal deformity, with or without cast; 7 to 12 vertebral segments
22804	Arthrodesis, posterior, for spinal deformity, with or without cast; 13 or more vertebral segments
22808	Arthrodesis, anterior, for spinal deformity, with or without cast; 2 to 3 vertebral segments
22810	Arthrodesis, anterior, for spinal deformity, with or without cast; 4 to 7 vertebral segments
22812	Arthrodesis, anterior, for spinal deformity, with or without cast; 8 or more vertebral segments
22818	Kyphectomy, circumferential exposure of spine and resection of vertebral segment(s) (including body and posterior elements); single or 2 segments
22819	Kyphectomy, circumferential exposure of spine and resection of vertebral segment(s) (including body and posterior elements); 3 or more segments
22849	Reinsertion of spinal fixation device
22850	Removal of posterior nonsegmental instrumentation (e.g., Harrington rod)
22851	Application of intervertebral biomechanical device(s) (e.g., synthetic cage(s), methylmethacrylate) to vertebral defect or interspace (List separately in addition to code for primary procedure)
22852	Removal of posterior segmental instrumentation

© 2020 Warner et al. *JAMA Network Open.*

22853	Insertion of interbody biomechanical device(s) (e.g., synthetic cage, mesh) with integral anterior instrumentation for device anchoring (e.g., screws, flanges), when performed, to intervertebral disc space in conjunction with interbody arthrodesis, each interspace (List separately in addition to code for primary procedure)
22854	Insertion of intervertebral biomechanical device(s) (e.g., synthetic cage, mesh) with integral anterior instrumentation for device anchoring (e.g., screws, flanges), when performed, to vertebral corpectomy(ies) (vertebral body resection, partial or complete) defect, in conjunction with interbody arthrodesis, each contiguous defect (List separately in addition to code for primary procedure)
22855	Removal of anterior instrumentation
22857	Total disc arthroplasty (artificial disc), anterior approach, including discectomy to prepare interspace (other than for decompression), single interspace, lumbar
22858	Total disc arthroplasty (artificial disc), anterior approach, including discectomy with end plate preparation (includes osteophytectomy for nerve root or spinal cord decompression and microdissection); second level, cervical (List separately in addition to code for primary procedure)
22859	Insertion of intervertebral biomechanical device(s) (e.g., synthetic cage, mesh, methylmethacrylate) to intervertebral disc space or vertebral body defect without interbody arthrodesis, each contiguous defect (List separately in addition to code for primary procedure)
22862	Revision including replacement of total disc arthroplasty (artificial disc), anterior approach, single interspace; lumbar
22864	Removal of total disc arthroplasty (artificial disc), anterior approach, single interspace; cervical
22865	Removal of total disc arthroplasty (artificial disc), anterior approach, single interspace; lumbar
22867	Insertion of interlaminar/interspinous process stabilization/distraction device, without fusion, including image guidance when performed, with open decompression, lumbar; single level
22868	Insertion of interlaminar/interspinous process stabilization/distraction device, without fusion, including image guidance when performed, with open decompression, lumbar; second level (List separately in addition to code for primary procedure)
22869	Insertion of interlaminar/interspinous process stabilization/distraction device, without open decompression or fusion, including image guidance when performed, lumbar; single level
22870	Insertion of interlaminar/interspinous process stabilization/distraction device, without open decompression or fusion, including image guidance when performed, lumbar; second level (List separately in addition to code for primary procedure)
63170	Laminectomy with myelotomy (e.g., Bischof or DREZ type), cervical, thoracic, or thoracolumbar
63172	Laminectomy with drainage of intramedullary cyst/syrinx; to subarachnoid space
63173	Laminectomy with drainage of intramedullary cyst/syrinx; to peritoneal or pleural space
63180	Laminectomy and section of dentate ligaments, with or without dural graft, cervical; 1 or 2 segments

© 2020 Warner et al. *JAMA Network Open.*

63182	Laminectomy and section of dentate ligaments, with or without dural graft, cervical; more than 2 segments
63185	Laminectomy with rhizotomy; 1 or 2 segments
63190	Laminectomy with rhizotomy; more than 2 segments
63191	Laminectomy with section of spinal accessory nerve
63194	Laminectomy with cordotomy, with section of 1 spinothalamic tract, 1 stage; cervical
63195	Laminectomy with cordotomy, with section of 1 spinothalamic tract, 1 stage; thoracic
63196	Laminectomy with cordotomy, with section of both spinothalamic tracts, 1 stage; cervical
63197	Laminectomy with cordotomy, with section of both spinothalamic tracts, 1 stage; thoracic
63198	Laminectomy with cordotomy with section of both spinothalamic tracts, 2 stages within 14 days; cervical
63199	Laminectomy with cordotomy with section of both spinothalamic tracts, 2 stages within 14 days; thoracic
63200	Laminectomy, with release of tethered spinal cord, lumbar
63250	Laminectomy for excision or occlusion of arteriovenous malformation of spinal cord; cervical
63251	Laminectomy for excision or occlusion of arteriovenous malformation of spinal cord; thoracic
63252	Laminectomy for excision or occlusion of arteriovenous malformation of spinal cord; thoracolumbar

© 2020 Warner et al. *JAMA Network Open.*

eTable 2. Consortium to Study Opioid Risks and Trends (CONSORT) Definitions for Opioid Availability

**CONSORT Classification**	**Definition**
None	No opioid prescription availability in the 180-day period of interest (i.e. 180 days prior to surgery)
Short-Term	Opioid prescription availability for less than 90 days during the 180-day period of interest
Episodic	Opioid prescription availability for ≥90 but <120 days with <10 opioid prescriptions during the 180-day period of interest
Chronic	Opioid prescription availability >90 days with >10 prescriptions or opioid availability > 120 days during the 180-day period of interest

© 2020 Warner et al. *JAMA Network Open.*

eFigure. Study Population Flow Diagram

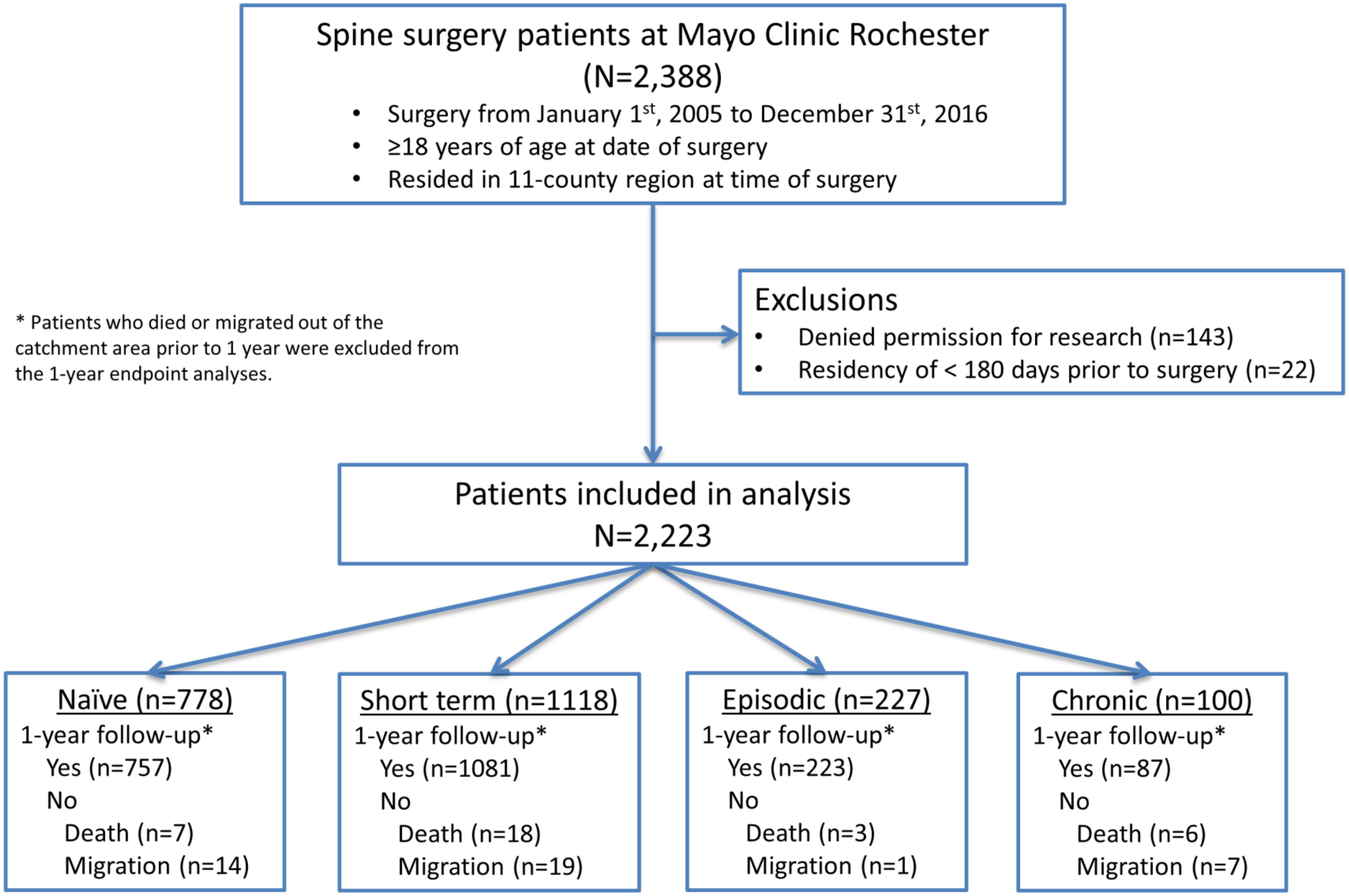

© 2020 Warner et al. *JAMA Network Open*.
